# Impaired perception of illusory contours and cortical hypometabolism in patients with Parkinson’s disease

**DOI:** 10.1016/j.nicl.2021.102779

**Published:** 2021-08-12

**Authors:** Toshiyuki Ishioka, Kazumi Hirayama, Yoshiyuki Hosokai, Atsushi Takeda, Kyoko Suzuki, Yoshiyuki Nishio, Yoichi Sawada, Nobuhito Abe, Etsuro Mori

**Affiliations:** a Department of Occupational Therapy, School of Health and Social Services, Saitama Prefectural University, Japan; b Department of Behavioral Neurology and Cognitive Neuroscience, Graduate School of Medicine, Tohoku University, Japan; c Department of Occupational Therapy, Yamagata Prefectural University of Health Science, Japan; d Department of Radiological Sciences, International University of Health and Welfare, Japan; e Department of Neurology, Sendai Nishitaga Hospital, Japan; f Department of Psychiatry, Tokyo Metropolitan Matsuzawa Hospital, Japan; g Department of Health and Welfare Science, Okayama Prefectural University, Japan; h Kokoro Research Center, Kyoto University, Japan; i Department of Behavioral Neurology and Neuropsychiatry, United Graduate School of Child Development, Osaka University, Japan

**Keywords:** Perception, Illusory contours, Parkinson's disease, Lateral occipital complex

## Abstract

Neuroimaging evidence suggests that areas of the higher-order visual cortex, including the lateral occipital complex (LOC), are engaged in the perception of illusory contours; however, these findings remain unsubstantiated by human lesion data. Therefore, we assessed the presentation time necessary to perceive two types of illusory contours formed by Kanizsa figures or aligned line ends in patients with Parkinson's disease (PD). Additionally, we used ^18^F-fluorodeoxyglucose positron emission tomography (FDG-PET) to measure regional cerebral glucose metabolism in PD patients. Although there were no significant differences in the stimulus durations required for perception of illusory contours formed by aligned line ends between PD patients and controls, PD patients required significantly longer stimulus durations for the perception of Kanizsa illusory figures. Difficulty in perceiving Kanizsa illusory figures was correlated with hypometabolism in the higher-order visual cortical areas, including the posterior inferior temporal gyrus. These findings indicate an association between dysfunction in the posterior inferior temporal gyrus, a region corresponding to a portion of the LOC, and impaired perception of Kanizsa illusory figures in PD patients.

## Highlights

• We assessed the perception of illusory contours in patients with PD.
• PD patients showed difficulty in perceiving Kanizsa illusory figures.
• Impaired perception of Kanizsa illusory figures was related to LOC hypometabolism.

## 1 Introduction

Illusory contours are apparent fine lines or edges that cannot be defined based on local luminance variations (Kanizsa et al., 1979). Previous neuroimaging studies have shown that portions of the early visual cortex, including V1/V2, are responsible for human perception of illusory contours (Ffytche and Zeki, 1996, Hirsch et al., 1995, Larsson et al., 1999, Seghier et al., 2000). In addition, several studies have demonstrated that parts of the higher-order visual cortex, including the lateral occipital cortex (LOC), a region known to be involved in object recognition, are also associated with the perception of illusory contours (Kruggel et al., 2001, Mendola et al., 1999, Murray et al., 2004, Pegna et al., 2002, Ritzl et al., 2003, Stanley and Rubin, 2003). These findings imply the critical role of feedback connections and top-down influences in the perception of illusory contours (for review, see Seghier and Vuilleumier, 2006).

Despite an accumulation of evidence from functional neuroimaging studies, there remains a dearth of neuropsychological evidence related to human perception of illusory contours. To date, several single-case neuropsychological studies have reported that impaired perception of illusory contours is caused by cerebral damage (Barton et al., 2007, de-Wit et al., 2009, Kartsounis and Warrington, 1991, Ricci et al., 1999, Vecera and Behrmann, 1997). However, these case reports have provided only inconclusive evidence on the exact neural correlates of the impaired perception of illusory contours due to limited generalizability. In addition, although several studies have examined the perception of illusory contours in groups of patients with different disorders (Fierro et al., 2003, Grabowska et al., 2001, Hamsher, 1978, Ota et al., 2015, Stroganova et al., 2007, Vuilleumier and Landis, 1998, Vuilleumier et al., 2001, Wasserstein et al., 1987), the findings from these studies have been diverse, and direct evidence of specific regional contributions to the perception of illusory contours is still lacking.

Therefore, in this study, we sought to identify regions involved in the perception of illusory contours from lesion data derived from a sizable series of patients with Parkinson's disease (PD), a neurological disorder known to cause deficits in a wide range of visual processes. The deficits in PD patients' visual processing are known to be derived not only from impaired retinal processing but also from impaired cortical processing (for review, see Bodis-Wollner, 2003). In addition, reductions in regional cerebral glucose metabolism and blood flow in posterior brain regions, including the visual cortices, have been repeatedly reported in PD patients (Abe et al., 2003, Firbank et al., 2003, Hosokai et al., 2009, Ishioka et al., 2011, Lyoo et al., 2010, Nishio et al., 2018, Nishio et al., 2017, Uchiyama et al., 2015). Critically, there are individual differences in both cognitive deficits and patterns of neurodegeneration in PD patients, allowing us to identify the link between impaired visual processing and regional dysfunction. Thus, neuropsychological investigations in PD patients are thought to provide unique evidence on neural correlates of perception of illusory contours.

The aim of this study was to test the hypothesis that PD patients show an impaired ability to perceive illusory contours associated with dysfunction in the higher-order visual cortex, including the LOC (Hosokai et al., 2009, Ishioka et al., 2011, Nishio et al., 2018, Nishio et al., 2017), which is located in the ventral form-processing stream. To this end, we assessed the presentation times necessary to perceive illusory contours in PD patients and healthy controls (HCs). Specifically, we prepared the stimuli for illusory contours formed by Kanizsa figures and by aligned line ends, and these stimuli were used to explore potential differences in perception of these two types of illusory contours. We also conducted ^18^F-fluorodeoxyglucose positron emission tomography (FDG-PET) imaging with a voxel-by-voxel, whole-brain analysis to effectively assess the relationship between an impaired ability to perceive illusory contours and decreased regional cerebral resting glucose metabolism in PD patients.

## 2 Methods

### 2.1 Subjects

Study participants included 42 idiopathic PD patients and 20 HCs matched for age, sex, educational attainment, visual acuity, and score on the Mini-Mental State Examination (MMSE). The demographic and clinical characteristics of the PD patients and HCs are shown in Table 1. All PD patients were selected from among those participating in a longitudinal cohort study of PD at Tohoku University Hospital (Baba et al., 2017, Baba et al., 2012, Shoji et al., 2014). HCs without histories of neurological or psychiatric diseases were recruited from local communities via an advertisement. The diagnosis of PD was made by board-certified neurologists based on the United Kingdom PD Society Brain Bank criteria (Gibb and Lees, 1988). Patient motor symptoms were evaluated using Hoehn-Yahr staging (Hoehn and Yahr, 1967) and the Unified Parkinson's Disease Rating Scale (UPDRS) motor part (Fahn and Elton, 1987).

**Table 1:** Demographic and clinical characteristics of PD patients and HCs.

Variables	PD	HC	*p*-values
	n = 42	n = 20	
Age (mean ± SD years)	65.0 ± 6.5	65.9 ± 5.6	0.58
Sex (female/male)	23/19	10/10	0.73
Education (mean ± SD years)	11.8 ± 2.3	11.2 ± 2.2	0.29
Median best-corrected visual acuity (range)	1.0 (0.7 – 1.0)	1.0 (0.7 – 1.0)	0.55
MMSE (mean ± SD; max. 30)	27.8 ± 2.0	28.5 ± 1.6	0.16
Hooper Visual Organization Test (mean ± SD)	18.9 ± 3.9 (n =31)	19.9 ± 4.2	0.37
Disease duration (mean ± SD years)	4.7 ± 3.9	–	–
Onset age (mean ± SD years)	60.2 ± 6.7	–	–
Median Hoehn and Yahr (range)	2.5 (1.0 – 3.0)	–	–
UPDRS Ш (mean ± SD)	19.2 ± 7.8	–	–
Daily levodopa equivalent dosage (mean ± SD mg)	559.0 ± 633.5	–	–
CDR (0/0.5)	33/9	–	–

CDR = Clinical Dementia Rating, HCs = healthy controls, MMSE = Mini-Mental State Examination, PD = Parkinson’s disease, SD = standard deviation, UPDRS = Unified Parkinson’s Disease Rating Scale, – no data. *T*-tests were used except for sex ratio (chi-square test) and best-corrected visual acuity (Mann-Whitney test).

The inclusion criteria for participation in this study were as follows: (1) age between 50 and 80 years at study initiation; (2) age at onset of PD over 40 years; (3) Hoehn and Yahr stage of 1 to 3; (4) no ocular disease; and (5) best-corrected visual acuity of 0.7 or better. The exclusion criteria were as follows: (1) complications due to other neurological or psychiatric diseases; (2) magnetic resonance imaging (MRI) evidence of focal brain lesions; or (3) dementia, i.e., stage 1 or higher according to Clinical Dementia Rating (CDR) (Hughes et al., 1982). Eleven patients with PD were de novo, and the remainder were treated with antiparkinsonian drugs, including five patients who were given trihexyphenidyl hydrochloride. None of the patients had any visual hallucinations during the month preceding participation in this study based on the Neuropsychiatric Inventory (NPI) (Cummings et al., 1994). None of the PD patients or HCs suffered from congenital achromatopsia.

All PD patients underwent PET scanning; however, control subjects were not scanned. Instead, normative PET data were used from another group consisting of 14 healthy participants without psychiatric or neurological diseases (7 females and 7 males; mean age: 64.0 ± 4.2 years; mean educational level: 12.3 ± 2.5 years) (Hosokai et al., 2009). There were no significant differences in age, sex, or educational attainment between the PD patients and these healthy participants (all *p*-values *> 0.1*). These normative data were used to create a mask image of brain regions demonstrating hypometabolism in PD patients. All PET images were obtained using the same machine and conditions.

After the subjects and their relatives had been given a complete description of the study, all participants provided written informed consent. The study was approved by the ethical committee of Tohoku University and was conducted in accordance with the Declaration of Helsinki.

### 2.2 Experimental tasks

**Fig. 1:**
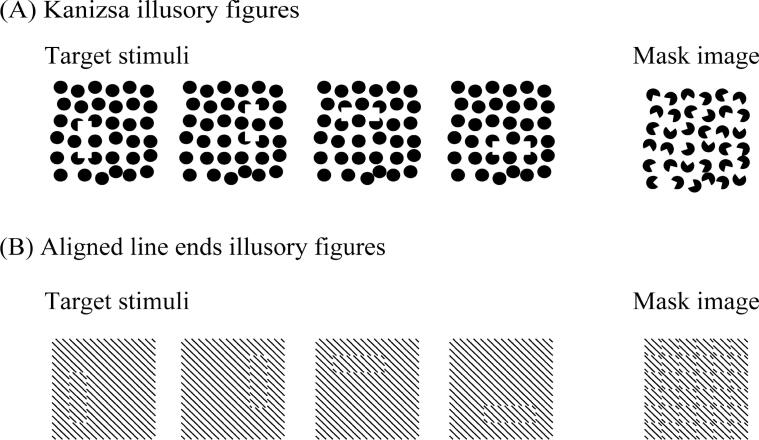
The four types of target stimuli and the mask image used in experimental tasks. (A) Illusory contours formed by Kanizsa figures. (B) Illusory contours formed by aligned line ends.

We prepared two different tasks to evaluate the perception of illusory contours formed by either Kanizsa figures or aligned line ends (Fig. 1). In the Kanizsa task, the target stimulus was a rectangular illusory figure formed by four Kanizsa-type inducers (incomplete black circles, “pac-men”) on a white background that included many complete black circles. There were four types of illusory rectangular target stimuli and one mask image. The four illusory rectangular targets included two with long horizontal sides and two with long vertical sides, each occluding two black circles. The support ratios (Shipley and Kellman, 1992) of the four illusory rectangular long sides were between 0.3 and 0.5. In the task involving illusory contours formed by aligned line ends, the target stimulus was a rectangular illusory figure induced by phase-shifted, abutting line gratings, which created oblique line segments out of alignment with oblique line segments in the background. For this task, there were also four types of target stimuli and one mask image. Targets of the displaced-grating illusory contours consisted of two rectangular targets with longer horizontal lines and two rectangular targets with longer vertical lines.

For each task, all stimuli were presented on a 17-inch display and controlled by a personal computer in a standardized, darkened environment. Targets and mask stimuli were presented at 75 cm, subtending 6° of the visual angle. To minimize the effects of impaired contrast sensitivity in PD patients, all targets and mask stimuli for each task consisted of achromatic images with a prominent contrast of 0.996, i.e., luminances of 0.27 cd/m^2^ for the black lines of each drawing and 136.7 cd/m^2^ for the white background. The contrast of these stimuli was more significant than the contrast of the visual acuity chart used in the present study (i.e., the contrast of the visual acuity chart was only 0.896).

**Fig. 2:**
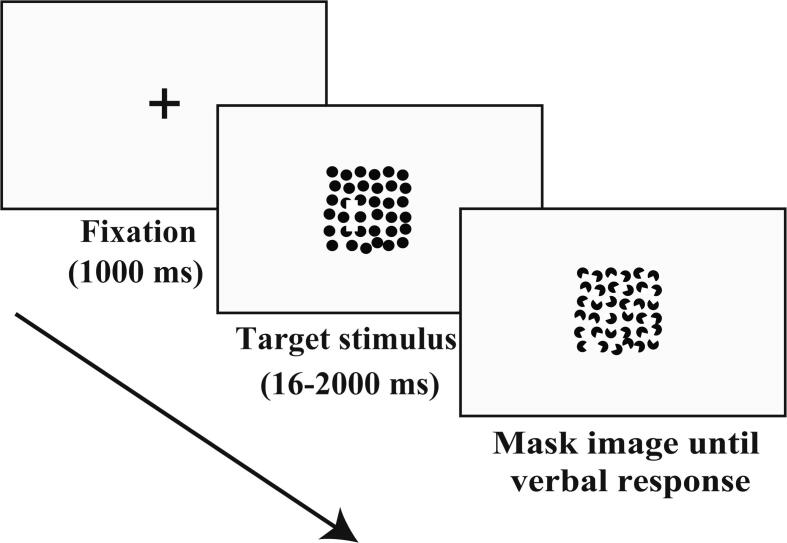
Schematic illustration of an example trial. Each trial started with the presentation of a fixation cross, which appeared 1000 ms before the target stimulus. The duration of target stimulus presentation varied according to the time required, as estimated by the best-parameter estimation by sequential testing (PEST) algorithm (16 – 2000 ms). Each target stimulus was followed by a mask image, which was displayed until the subject gave a verbal response.

The time sequence of stimulus presentation is represented in Fig. 2. Each trial started with the appearance of a fixation cross for a duration of 1000 ms. Subsequently, the target stimulus appeared for a variable period of time (16–2000 ms) followed by a mask image. The mask image was presented to suppress any afterimages until the subject responded. The subjects were instructed to direct their gaze at the fixation cross in the center of the display. After the fixation cross had disappeared, a target stimulus appeared, and subjects were instructed to verbally respond to each stimulus in an alternative-choice paradigm, i.e., stating whether a target of rectangular shape was horizontally or vertically long. The experimenter pressed a button based on whether the subject had given an accurate or an inaccurate response, after which the next fixation interval and target presentation occurred. The total experimental time of the illusory contour perception task was less than three minutes. At the beginning of each task, subjects performed a practice session (four trials with each target stimulus) to familiarize themselves with the task.

To minimize the effects of motor symptoms in PD patients, no time limit was set for responding to each trial, and reaction times were not measured. Instead, as has previously been used in a study of preattentive visual processing in PD patients (Lieb et al., 1999), our outcome measure was the presentation time necessary to perceive a target stimulus during each task. The presentation time of a target stimulus, which is considered to be the perceptual threshold, was controlled by the best-parameter estimation by sequential testing (PEST) algorithm (Lieberman and Pentland, 1982). This procedure assumes that performance will improve monotonically as stimulus duration increases. For our tasks, stimulus duration was plotted on a logarithmic time (linear angle) scale, and the best-fitting curve was a sigmoidal logistic function. The best-PEST procedure used a maximum likelihood criterion for determining the stimulus duration of the next trial. The results of all preceding trials were accumulated to calculate the presentation time that was the most likely estimate of the putative threshold. The duration of stimulus presentation was therefore determined for each subject individually. Based on pilot experiments, 40 trials were presented for each task, while the best-PEST “homed-in” on the threshold. While Lieb et al. (1999) set the threshold as 62.5% correct in the task with four alternatives (i.e., chance level = 25%), we set the threshold as 75% correct in the task with two alternatives (i.e., chance level = 50%) based on procedures in previous studies (Madigan and Williams, 1987, Pentland, 1980). The threshold time for each subject was calculated from the 75th percentile of the sigmoid function that was estimated by a significant model (log-likelihood ratio tests; *p* < .05) based on the generalized linear model (GLM) for binary responses.

### 2.3 PET data acquisition

In PD patients, the regional cerebral metabolic rate of glucose utilization at rest was measured using FDG and PET. All patients fasted for at least 5 h before scanning and were given 185 – 218 MBq of FDG intravenously 1 h before scanning. The subjects were studied under resting conditions with their eyes closed and ears unplugged. PET images were obtained using a biograph DUO PET/computed tomography (CT) scanner (Siemens Medical System, Inc., USA) over 10 min. The in-plane and axial resolutions of the scanner were 3.38 and 3.38 mm full-width at half maximum (FWHM), respectively. Image reconstruction was performed using ordered subset expectation maximization with 16 subsets and a six-iteration reconstruction algorithm (Gaussian filter; filter FWHM: 2.0 mm), displayed in a 256 × 256 matrix (pixel size: 1.33 × 1.33 mm with a slice thickness of 2.0 mm). Attenuation correction was performed with the built-in CT scan. The interval between neuropsychological testing and PET scanning was less than 4 weeks.

### 2.4 Statistical analysis

Statistical analysis was conducted using Jamovi (Version 1.6.6.0-win64). Student's/Welch's *t*-tests were used to analyze performance on experimental tasks and demographic and neuropsychological data, except for the sex ratio (chi-square test) and the best-corrected visual acuity (Mann-Whitney test). Moreover, analyses of covariance (ANCOVA), with the MMSE scores as nuisance variables to control for general cognitive dysfunction, were conducted to compare task performance related to illusory contours between the PD and HC groups. Pearson's product-moment correlation coefficients or Spearman rank-correlation coefficients were calculated to analyze the relationships between performance on tasks and demographic or clinical characteristics in the PD and HC groups, respectively.

PET images were analyzed using Statistical Parametric Mapping version 12 software (SPM12; Wellcome Department of Imaging Neuroscience, London, U.K.) implemented in MATLAB 9.0 (The Math Works Inc., MA, USA). PET images were normalized to the FDG template based on the Montreal Neurological Institute (MNI) reference brain (resampled voxel size, 2 × 2 × 2 mm^3^) and were smoothed using an isotropic 10-mm FWHM Gaussian kernel. Each voxel count was normalized to the total count of the brain using proportional scaling to reduce between-subject variation in global metabolic rates.

For each experimental task, threshold times to perceive illusory contours of PD patients were entered as covariates of interest in the correlation analyses to determine regions showing decreased metabolism associated with poor performance (i.e., an increase in the presentation time necessary to perceive a target stimulus). To confine our analysis to regions showing hypometabolism in PD patients relative to healthy participants, PET data obtained from our sample of 42 PD patients were contrasted with data obtained from a group of 14 healthy participants (who did not participate in the present neuropsychological study), and a resulting map with a liberal threshold (*p* < .05, uncorrected) was used for masking in correlation analyses. Age, sex, effects of medication (i.e., daily levodopa equivalent dose), the severity of extrapyramidal features (i.e., UPDRS motor part score), and general cognitive impairment (i.e., MMSE score) – all of which are possible confounding factors for regional metabolism – were controlled by entering these variables into the model. For the whole-brain analysis, the significance threshold was set at a *p*-value of <0.001 at the voxel level (uncorrected for multiple comparisons), and clusters were considered significant if they passed a cluster-level threshold of *p* < .05 after familywise error (FWE) correction. In separate analyses, we also used a threshold-free cluster enhancement (TFCE) technique to reduce possible false positive voxels and “cluster leaking” issues in cluster-level inferences (Eklund et al., 2016). The TFCE was implemented in an SPM Toolbox with default settings (Smith and Nichols, 2009).

To further analyze the relationships between the impaired perception of illusory contours and LOC dysfunction, a region involved in early perceptual processing for detecting a figure induced by illusory contours (Shpaner et al., 2009), we conducted region of interest (ROI) analyses. For the unbiased ROI analysis to minimize double-dipping (Kriegeskorte et al., 2009), we created the LOC ROI as the overlapping regions of (A) the cluster with a radius of 10 mm from the peak coordinate of the LOC in the previous literature (see Lerner et al., 2002) using WFU_Pickatlas 3.0.5 and (B) the statistical maps based on the automated meta-analysis of the NeuroSynth database (taken from a keyword search “object recognition”, http://www.neurosynth.org). The FDG uptake values in the above LOC ROI were obtained from the PD patients and entered into the correlation analysis to examine the association between decreased regional cerebral glucose metabolism and the presentation time to perceive illusory contours.

## 3 Results

### 3.1 Experimental tasks

To calculate the threshold time to perceive illusory contours for each participant, we first conducted the log-likelihood ratio test with GLM for binary responses. This analysis showed that two subjects (one PD patient and one healthy control) did not reach a significant model for the Kanizsa-type illusory contour (*p* > .05) and that a different PD patient did not reach a significant model for the illusory contour induced by aligned line edges (*p* > .05). The data from these participants were excluded from the analyses.

Table 2 lists the results of the experimental tasks in PD patients and HCs. We found a significant difference in the presentation time necessary to perceive Kanizsa-type illusory contours between the PD patients (n = 41) and HCs (n = 19) (*t*_(56.5)_ = 3.70, *p* < .001; Cohen's *d* = 0.771, 95% confidence interval (CI): 0.198 to 1.344). However, we failed to detect a significant difference in the presentation time necessary to perceive illusory contours induced by aligned line ends between the PD patients (n = 41) and HCs (n = 20) (*t*_(59.0)_ = 1.90, *p* = .062; Cohen's *d* = 0.519, 95% CI: −0.046 to 1.07). While an ANCOVA controlling for MMSE scores revealed a significant difference in threshold times on Kanizsa-type stimuli between the two groups (estimated marginal mean ± SE, 95% CI; PD: 192 ± 9.37, 173 to 211, HC: 150 ± 13.84, 123 – 178; F_(1, 57)_ = 6.15, *p* = .016), it revealed no significant difference in threshold time on aligned line ends type stimuli (estimated marginal mean ± SE, 95% CI; PD: 172 ± 9.46, 153 to 191, HC: 140 ± 13.63, 113 to 168; F_(1, 58)_ = 3.543, *p* = .065).

**Table 2:** Threshold time to perceive illusory contour task in PD patients and HCs.

Threshold time (mean ± SD ms)	PD	HC		*t*-test	ANCOVA (controlling for MMSE scores)			
			*t*-value	Cohen's d (95% CI)	*p*-value	*F*-value	η^2^	*p*-value
(A) Kanizsa figures	194.0 ± 71.1 (n = 41)	147.0 ± 26.9 (n = 19)	3.70	0.771 (0.198 to 1.344)	< 0.001	6.120	0.094	0.016
(B) Aligned line ends	172.0 ± 66.9 (n = 41)	141.0 ± 40.7 (n = 20)	1.90	0.519 (−0.046 to 1.070)	0.062	3.543	0.058	0.065

ANCOVA = analysis of covariance, CI = confidence interval, HCs = healthy controls, MMSE = Mini-Mental State Examination, PD = Parkinson's disease, SD = standard deviation.

The results of the correlation analyses between the threshold times for each experimental task and demographic/clinical data in PD patients are summarized in Table 3. The threshold time to perceive Kanizsa illusory figures was significantly correlated with age at disease onset, whereas the threshold time to perceive illusory contours formed by aligned line ends was significantly correlated with chronological age, age at disease onset, and UPDRS III score.

**Table 3:** Correlations between the threshold time to perceive illusory contours and demographic/clinical characteristics in PD patients.

*r* (95% CI) *p*-value	Age	Education	Visual acuity	Disease duration	Onset age	UPDRS III	Levodopa	MMSE
(A) Kanizsa figures	*r* = 0.28 (−0.03 to 0.54)	*r* = −0.11 (−0.41 to 0.20)	*r_s_* = −0.21	*r* = −0.11 (−0.41 to 0.20)	***r* = 0.34 (0.03 to 0.58)**	*r* = 0.12 (−0.20 to 0.41)	*r* = 0.22 (−0.09 to 0.50)	*r* = −0.25 (−0.52 to 0.06)
(n = 41)	*p* = .072	*p* = .486	*p* = .144	*p* = .490	***p* = .032**	*p* = .461	*p* = .164	*p* = .115
(B) Aligned line ends	***r* = 0.43 (0.14to0.65)**	*r* = −0.11 (−0.40 to 0.21)	*r_s_* = −0.24	*r* = 0.02 (−0.29 to 0.33)	***r* = 0.40 (0.10 to 0.63)**	***r* = 0.35 (0.05 to 0.60)**	*r* = 0.18 (−0.13 to 0.46)	*r* = 0.006 (−0.25 to 0.36)
(n = 41)	***p* = .005**	*p* = .508	*p* = .125	*p* = .964	***p* = .010**	***p* = .024**	*p* = .253	*p* = .699

MMSE = Mini-Mental State Examination, PD = Parkinson's disease, UPDRS = Unified Parkinson's Disease Rating Scale. Pearson's product-moment correlation coefficients were used except for visual acuity (Spearman rank-correlation coefficient). Bold indicates significant correlations.

The results of correlation analyses between the threshold times for each experimental task and demographic/neuropsychological data in HCs are shown in Supplementary Table 1. There was a significant positive correlation between the threshold time to perceive illusory contours formed by aligned line ends and age (*r* = 0.479, *p* = .0032, 95% CI: 0.047 to 0.865). No significant correlation was observed between the perception of Kanizsa illusory contours and demographic/neuropsychological data (all *p*-values > 0.05).

### 3.2 Voxel-based cognitive-metabolic correlation analyses

**Fig. 3:**
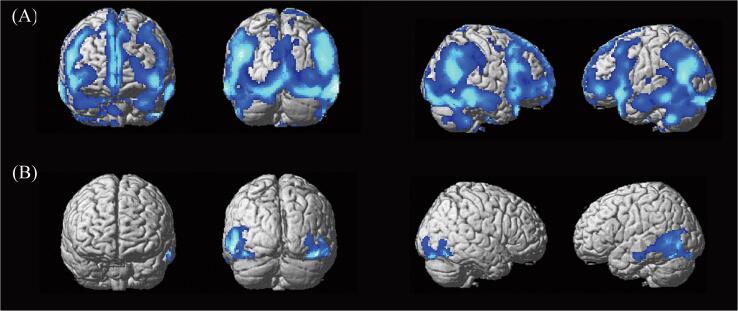
(A) Brain regions showing hypometabolism in PD patients compared with healthy participants. Note that the statistical threshold was relatively liberal in this group comparison (*p* < .05, uncorrected), since this analysis was done only for generating the mask image included in the cognitive-metabolic correlation analysis within the group of PD patients. The regions are displayed on a surface-rendered standard brain. (B) Brain regions showing significant correlations between the threshold time to perceive Kanizsa illusory figures and regional cerebral glucose metabolism in PD patients. Note that the results were masked with the above contrast of the healthy participants versus the PD patients to confine our analysis to the regions showing hypometabolism in the PD patients. The possible confounding effects of age, sex, daily levodopa equivalent dose, UPDRS motor part scores, and MMSE scores were also controlled for.

We first conducted whole-brain correlation analyses between the threshold time to perceive a figure induced by illusory contours and resting regional cerebral glucose metabolism in PD patients. Since we used threshold time as the outcome measure for task performance, regions showing negative correlations reflected lesions responsible for the impaired perception of illusory contours. This analysis revealed that the threshold time to perceive Kanizsa illusory figures was negatively correlated with resting FDG uptake values in the left inferior temporal gyrus and right inferior occipital gyrus extending to the inferior temporal gyrus (Table 4 and Fig. 3B). The threshold time to perceive illusory contours formed by aligned line ends was not correlated with regional metabolic rates in any brain region. Even when we conducted this analysis without inclusive masking obtained from group comparison, the results on Kanizsa illusory figures virtually remained unchanged (Supplementary Table 2 and Supplementary Fig. 1).

**Table 4:** Brain regions showing a significant correlation between the threshold time to perceive Kanizsa illusory figures and regional cerebral glucose metabolism in PD patients.

	Cluster level		Peak voxel				
Brain region			Coordinates (mm)			*p* (uncorrected)	*Z*-score
	*p* (FWE-corrected)	Cluster size	x	y	z		
Left inferior temporal gyrus (extending to the middle temporal gyrus and inferior occipital gyrus)	0.002	1392	−56	−66	−10	< 0.001	4.80
			−54	−62	4	< 0.001	4.26
			−64	−18	−20	< 0.001	4.08
Right inferior occipital gyrus (extending to the inferior temporal gyrus and occipital fusiform gyrus)	0.037	763	46	−86	−16	< 0.001	4.57
			60	−66	−14	< 0.001	3.93
			52	−68	0	< 0.001	3.91

FWE = familywise error, PD = Parkinson's disease.

We also conducted the SPM analysis with TFCE. While the results on Kanizsa illusory figures remained virtually unchanged from the original SPM results (Supplementary Table 3 and Supplementary Fig. 2A), the threshold time to perceive illusory contours formed by aligned line ends was significantly correlated with regional metabolic rates in the bilateral posterior middle temporal gyri extending to inferior and posterior regions and the middle occipital gyrus extending to the inferior parietal lobule (Supplementary Table 3 and Supplementary Fig. 2B).

**Fig. 4:**
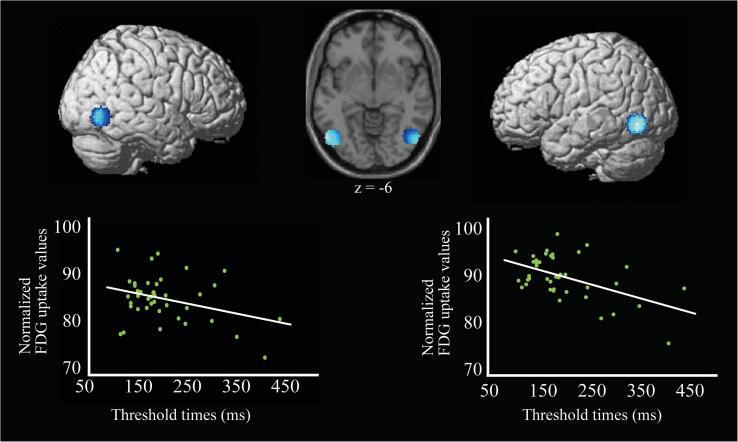
Results of LOC ROI analysis displayed on an average single-subject T1 image (z = −6) and surface-rendered standard brain. Scatter plots indicate significant negative correlations between the threshold time to perceive Kanizsa illusory figures and FDG uptake values in the right LOC (*r* = −0.339, 95% CI: −0.585 to −0.035, *p* = .030) and left LOC (*r* = −0.509, 95% CI: −0.706 to −0.239, *p* < .001).

Next, we conducted an ROI analysis with the LOC regarding performance in the perception of Kanizsa illusory contours. Note that the ROI used here was the overlapping region of the spherical ROI based on the past literature (Lerner et al., 2002) and the meta-analysis map of regions associated with object recognition, thus avoiding the problems associated with double dipping (see above). As shown in Fig. 4, the threshold time on Kanizsa illusory stimuli was negatively correlated with the FDG uptake values in the right LOC (*r* = −0.339, 95% CI: −0.585 to −0.035, *p* = .030) and with those in the left LOC (*r* = −0.509, 95% CI; −0.706 to −0.239, *p* < .001).

## 4 Discussion

This study demonstrated that the perception of Kanizsa-type illusory contours, as indexed by the presentation time necessary to perceive a target stimulus, was impaired in PD patients relative to HCs. Furthermore, FDG-PET imaging revealed that the impaired perception of Kanizsa-type illusory contours was significantly correlated with decreased metabolic rates in the posterior inferior temporal gyrus in PD patients, regardless of their age, sex, or other possible confounding factors. Notably, the posterior inferior temporal gyrus corresponds to a portion of the LOC, a region located in the higher-order visual cortex of the ventral form-processing stream. The present study provides direct neuropsychological evidence that the LOC is closely linked to human perception of Kanizsa-type illusory contours.

Most importantly, the present neuropsychological evidence substantiates functional neuroimaging findings that the higher-order visual cortex is associated with the perception of illusory contours (Kruggel et al., 2001, Mendola et al., 1999, Murray et al., 2004, Pegna et al., 2002, Ritzl et al., 2003, Shpaner et al., 2013, Stanley and Rubin, 2003). Lesion design is a crucial methodology for demonstrating the involvement of a brain region in a particular cognitive process (Rorden and Karnath, 2004). Resting-state studies of the glucose metabolic rate using FDG-PET are considered to be particularly useful in the context of neuropsychological investigations in PD patients (Abe et al., 2009, Ishioka et al., 2011, Mentis et al., 2002, Nishio et al., 2018, Nishio et al., 2017, Uchiyama et al., 2015) because the regional metabolic rate is a marker of integrated local synaptic activity and is sensitive to both direct neuronal/synaptic damage and secondary functional disruption at synapses distant from the primary site of pathology (Magistretti et al., 1999).

The present results are consistent not only with functional neuroimaging findings but also with the results of previous studies using interventional and neuropsychological techniques. For example, using repetitive transcranial magnetic stimulation, Brighina et al. (2003) reported the critical role of the right extrastriate cortex in the perception of Kanizsa illusory figures. Furthermore, impaired perception of illusory contours has been reported in a patient with Balint's syndrome and bilateral occipitotemporal lesions (Barton et al., 2007) and a patient with bilateral damage to the lateral occipital cortex with a spared V1 region (de-Wit et al., 2009). As such, the present study, which used an unbiased voxel-by-voxel, whole-brain analysis of lesion data derived from a large number of patients, provides further evidence for the necessary involvement of the LOC in illusory contour perception.

The visual system is widely believed to be organized into two segregated pathways: the ventral visual stream, which is involved in object vision, and the dorsal visual stream, which is involved in spatial vision (Mishkin et al., 1983, Ungerleider and Haxby, 1994). The bilateral posterior inferior temporal gyri, which showed hypometabolism in the present study, has been demonstrated to be a part of the LOC in the ventral visual stream (Grill-Spector, 2003). This finding is consistent with our *a priori* hypothesis and indicates that the process of shape computation in the LOC (Gilaie-Dotan et al., 2002, Kourtzi and Kanwisher, 2000, Malach et al., 1995, Shpaner et al., 2009) might be crucial for the perception of illusory contours. In our tasks, the subjects were asked to indicate whether the target stimulus derived from illusory contours had a horizontally or vertically longer rectangular shape. Therefore, dysfunction in the LOC is likely to affect patient performance when extracting shape information formed by illusory contours.

The fact that the PD patients had an increased threshold time to perceive a target stimulus on Kanizsa illusory contours does not necessarily indicate that they cannot perceive a target stimulus at all. The prolonged threshold time on Kanizsa illusory contours in this study is consistent with the two previous studies showing deficits in visuoperception in PD patients characterized by prolonged presentation times (Johnson et al., 2004, Lieb et al., 1999). Notably, Lieb et al. (1999) found a prolonged threshold time for two types of stimuli: one was a target that consisted of differentially oriented line segments into a background, while the other was a target “L” among many “+” distractors in a task assessing preattentive visual processing. However, the presentation time to perceive a target “L” among “T” distractors “T” that requires attention, such as visual scanning, was comparable to that of controls (Lieb et al., 1999). Another study (Johnson et al., 2004) found a prolonged presentation time for a task in which participants had to identify the length of two vertical lines joined at the top by a horizontal line segment. In the present study, we interpreted the prolonged presentation time of the Kanizsa stimuli as resulting from a similar decline in preattentive visual processing. Based on a model suggesting that the processing of illusory contours involves top-down processing from the LOC to lower-order visual cortex, we propose that PD patients can perceive a figure induced by illusory contours when provided with sufficient presentation time because of the relatively intact ability of delayed processing in lower-order visual cortex.

In addition to the LOC, we also revealed significant correlations between the threshold time to perceive Kanizsa illusory figures and regional metabolism in the right fusiform gyrus located in the ventral visual stream and human MT+/V5 to anterior V3a located in the dorsal visual stream. The relationship between the right fusiform gyrus and the ability to perceive a figure induced by illusory contours is consistent with the results of activation studies using PET (Hirsch et al., 1995, Larsson et al., 1999). Human MT+/V5 and V3a are known to be associated with depth perception with slanted stereo surfaces (Welchman et al., 2005). The reduced processing ability of depth perception associated with dysfunctional human MT+/V5 and V3a may lead to a prolonged threshold time for Kanizsa-type stimuli in PD patients.

The difference in presentation time to perceive illusory contours formed by aligned line ends did not reach statistical significance between PD patients and HCs. However, by this, we do not wish to imply that the two types of illusory contour perceptions are supported by different neural mechanisms. Instead, we believe that the neural correlates of the two types of illusory contour perception largely overlap. That is, there might be no essential difference in the perception of two types of illusory contours, and the lack of difference in the aligned line ends task might have been due to lack of statistical power based on a relatively small sample size. This interpretation is supported by our findings that the threshold time to perceive illusory contours formed by aligned line ends showed a marginal difference between the two groups and that the FDG-PET analysis with the TFCE method detected a link between the threshold time and regional glucose metabolism in posterior cortical regions, including the LOC (Supplementary Fig. 2B). Alternatively, the impairment of illusory contour perception formed by aligned line ends might become more apparent with the progression of the disease. Therefore, future studies with larger sample sizes and more advanced PD groups may shed further light on this issue.

Two limitations of the present study warrant attention. First, while we attempted to minimize the effects of contrast sensitivity on task performance, we could not entirely rule out the possibility that the present results were confounded by defective contrast sensitivity in PD patients (cf., Uc et al., 2005). Second, since we did not identify the impairment of illusory contour perception at the individual level, the prevalence of impairment in PD patients remains unclear. Longitudinal studies with a larger number of patients will be necessary to follow the incidence of impaired illusory contour perception caused by LOC dysfunction.

## 5 Conclusions

In conclusion, this study revealed that PD patients show an impaired ability to perceive Kanizsa-type illusory contours associated with dysfunction in the higher-order visual cortex. Deficits in extracting shape information in the ventral stream resulting from LOC dysfunction are likely to contribute to impaired perception in PD patients. These neuropsychological findings substantiate functional neuroimaging findings and support the view that the higher-order visual cortex is responsible for the human perception of illusory contours.

## CRediT authorship contribution statement

**Toshiyuki Ishioka:** Conceptualization, Methodology, Formal analysis, Investigation, Data curation, Writing – original draft, Writing - review & editing, Visualization, Funding acquisition. **Kazumi Hirayama:** Conceptualization, Methodology, Investigation, Writing – original draft, Writing - review & editing. **Yoshiyuki Hosokai:** Software, Investigation, Formal analysis, Writing - review & editing. **Atsushi Takeda:** Resources, Investigation, Supervision. **Kyoko Suzuki:** Investigation, Supervision. **Yoshiyuki Nishio:** Investigation, Supervision. **Yoichi Sawada:** Investigation, Supervision. **Nobuhito Abe:** Writing – original draft, Writing - review & editing, Visualization. **Etsuro Mori:** Conceptualization, Methodology, Project administration, Supervision, Data curation, Writing - original draft.
