# Supplementary material for: Impaired perception of illusory contours and cortical hypometabolism in patients with Parkinson’s disease
Source: Neuroimage Clin. 2021 Aug 12;32:102779. doi: 10.1016/j.nicl.2021.102779 (PMC8385116; doi:10.1016/j.nicl.2021.102779)
Supplement: Supplementary data 1 [file mmc1.pdf]

Supplementary Table 1. Correlations between the threshold time to perceive illusory contours and demographic characteristics in HCs

***r* (95% CI)<br>*p*-value**	**Age**	**Education**	**Visual acuity**	**MMSE**
**(A) Kanizsa figures**	r = .23 (-.25 to .62)	r = -.03 (-.48 to .43)	r_s = -.03	r = -.18 (-.59 to .30)
(n = 19)	p = .35	p = .892	p = .920	p = .453
**(B) Aligned line ends**	**r = .47 (.05 to .76)**	r = -.07 (-.50 to .39)	r_s = -.02	r = -.17 (-.57 to .29)
(n = 20)	**p = .032**	p = .780	p = .944	p = .699

HCs = healthy controls, MMSE = Mini-Mental State Examination. Pearson's product-moment correlation coefficients were used except for visual acuity (Spearman rank-correlation coefficient). Bold indicates significant correlations. Note that one participant was excluded from the analysis (A) because an appropriate threshold time on the Kanizsa illusory contours task could not be obtained.

Supplementary Table 2. Brain regions showing a significant correlation between the threshold time to perceive Kanizsa illusory figures and regional cerebral glucose metabolism in PD patients without inclusive masking obtained from the group comparison of PD patients vs. HCs.

**Brain region**	**Cluster level**		**Peak voxel**				
			**Coordinates (mm)**				
	***p* (FWE-corrected)**	**Cluster size**	**x**	**y**	**z**	***p* (uncorrected)**	***Z*-score**
**Left inferior temporal gyrus**	**.002**	**2092**	-56	-66	-10	< .001	4.87
(extending to the middle temporal			-54	-62	4	< .001	4.35
gyrus and inferior occipital gyrus)			-64	-18	-18	< .001	4.20
**Right inferior occipital gyrus**	**.022**	**867**	46	-86	-16	< .001	4.56
(extending to the inferior temporal			52	-70	0	< .001	3.89
gyrus and fusiform gyrus)			60	-66	-14	< .001	3.87

FWE = familywise error, HCs = healthy controls, PD = Parkinson's disease.

Supplementary Table 3. Brain regions showing a significant correlation between the threshold time to perceive two types of illusory stimuli and regional cerebral glucose metabolism in PD patients in SPM analysis with TFCE.

Stimulus type	Brain region	equivalent cluster size	Coordinates (mm)			TFCE	*p* (FWE-corr)
			x	y	z		
**(A) Kanizsa figures**	**Left middle temporal gyrus** (extending to the inferior and superior temporal gyri, middle and inferior occipital gyri, and inferior parietal lobule)	3796	-56	-66	-10	1443.519	.001
			-54	-62	4	1325.391	.002
			-44	-62	-20	1163.916	.004
	**Right inferior occipital gyrus** (extending to the inferior and middle temporal gyri, fusiform gyrus, and occipital pole)	1094	46	-86	-16	1002.222	.008
			60	-66	-14	830.101	.017
			52	-70	-2	825.090	.017
**(B) Aligned line ends**	**Left middle temporal gyrus** (extending to the inferior temporal gyrus, middle and inferior occipital gyri, and angular gyrus)	984	-54	-54	-8	1145.290	.004
			-50	-64	4	1075.205	.005
			-40	-70	10	764.056	.02
	**Left middle occipital gyrus** (extending to the angular gyrus)	689	-34	-84	34	779.431	.019
			-30	-76	36	766.289	0.02

		-44	-78	36	761.767	.02
**Right angular gyrus** (extending to the middle and inferior temporal gyri, middle and inferior occipital gyri, and supramarginal gyrus)	1844	54	-66	2	774.387	.019
		54	-76	36	732.395	.023
		36	-74	38	693.755	.028
**Left inferior parietal lobule**	96	-52	-52	30	602.275	.042

FWE = familywise error, PD = Parkinson's disease, TFCE = threshold-free cluster enhancement, SPM = Statistical Parametric Mapping.

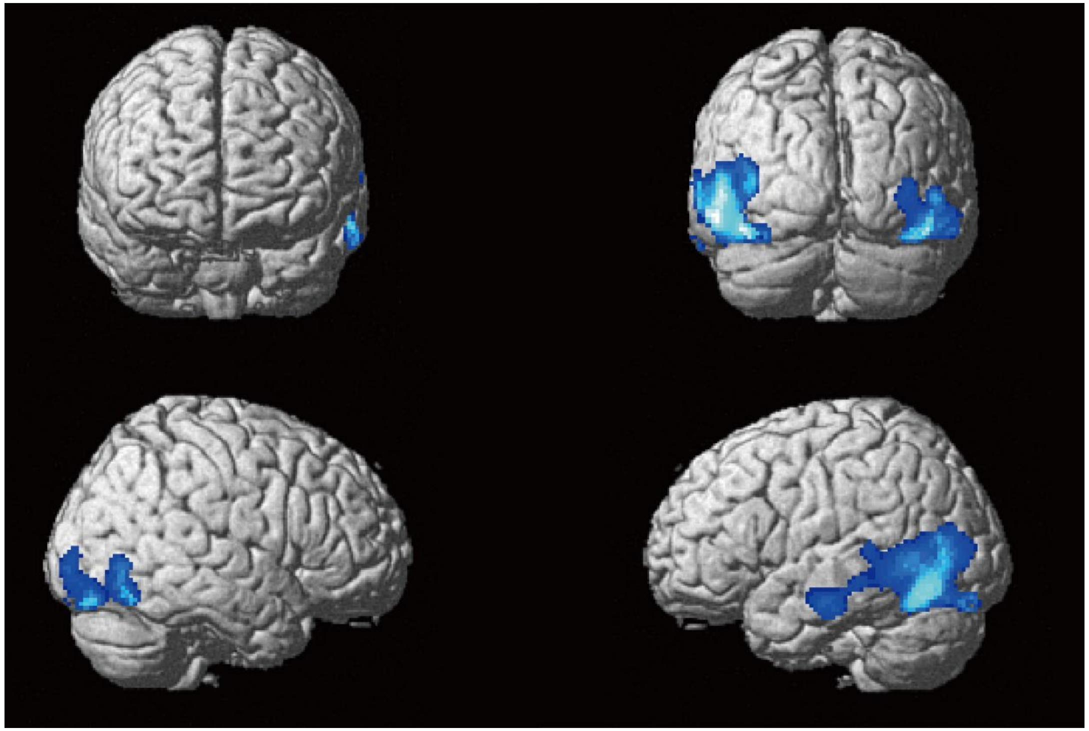

Supplementary Figure 1

Brain regions showing significant correlations between the threshold time to perceive Kanizsa illusory figures and the regional cerebral glucose metabolism in PD patients without the mask obtained from the group comparison of PD patients vs. HCs.

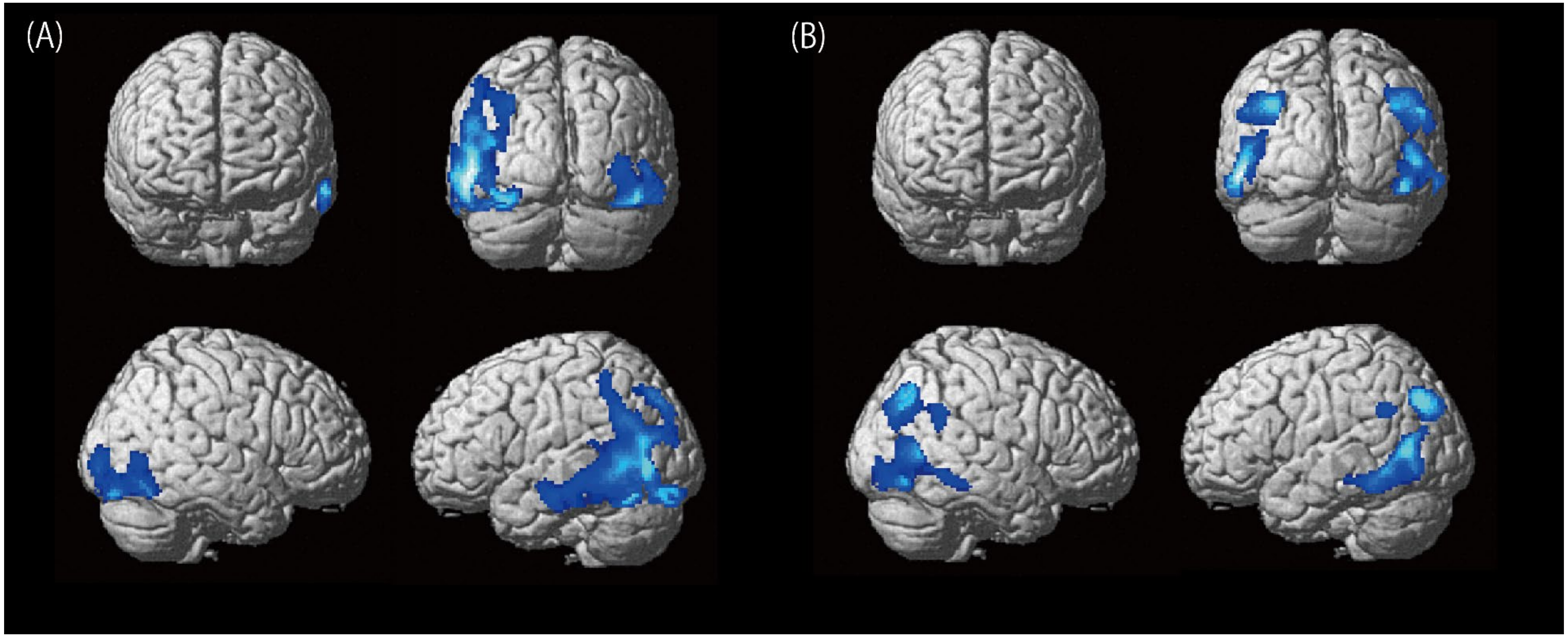

Supplementary Figure 2

Brain regions showing a significant correlation between the threshold time to perceive two types of illusory contours and regional cerebral glucose metabolism in PD patients in SPM analysis with TFCE (FWE $p < 0.05$). (A) Kanizsa figures and (B) aligned line ends.
